# *Cephalaria anamurensis* (Caprifoliaceae), a new species from south Anatolia, Turkey

**DOI:** 10.3897/phytokeys.65.8571

**Published:** 2016-06-15

**Authors:** Ramazan Süleyman Göktürk, Hüseyin Sümbül

**Affiliations:** 1 Department of Biology, Faculty of Science, Akdeniz University, 07058, Antalya, Turkey

**Keywords:** Cephalaria, New species, Taxonomy, Turkey

## Abstract

A new species, *Cephalaria anamurensis* (Caprifoliaceae) is described and illustrated from south Anatolia, Turkey. The species grows on steppe and stony places in Anamur (C4 Mersin province) in south Anatolia. Diagnostic morphological characters from closely similar taxa are discussed. The geographical distribution of the new species and two closely related species in Turkey are mapped.

## Introduction

The genus *Cephalaria* Schrad. ex Roem. & Schult. was first described by J.J. Roemer and J.A. Schultes (Roemer and Schultes 1818). It is distributed from Mediterranean area to west China, and some of the species are also found in southern Africa (Szabó 1940). The genus *Cephalaria* has long been regarded as belonging to the Dipsacaceae, whereas according to APG III it is included within the larger family Caprifoliaceae (Dipsacales) and consist of 100 species (Reveal and Chase 2011). In Turkey, the family Caprifoliaceae is represented by 12 genera, namely *Centranthus* DC. (3 spp.), *Cephalaria* (39 spp.), *Dipsacus* L. (5 spp.), *Knautia* L. (9 spp.), *Lonicera* L. (11 spp.), *Morina* L. (2 spp.), *Pterocaphalus* Adans. (9 spp.), *Scabiosa* L. (32 spp.), *Succisa* Haller (1 sp.), *Tremastelma* Rafin. (1 sp.), *Valeriana* L. (14 spp.) and *Valerianella* Mill. (31 spp.) (Güner et al. 2012).

In July 2015, the authors collected a specimen of *Cephalaria* from the Taurus Mountains (Mersin province) during fieldwork for Expo Antalya 2016 natural plant supply project. Fruiting material was gathered in the same area in September 2015 by the authors. This specimen has been compared to many specimens including two supposedly closely related species in the Herbaria of Akdeniz University Herbarium, ANK, GAZI and HUB, records in the literature (Szabó 1940, Göktürk et al. 2012, Göktürk and Sümbül 2014) and consulting floras of Turkey and neighboring countries (Bobrov 1957, Matthews 1972, Ferguson 1976, Feinbrun-Dothan 1978, Matthews 1988, Lack 1991). Successful efforts have also been made to find additional locations in this vicinity where this novel plant may be located. After comparison with material of morphologically similar taxa, we concluded that these specimens represent a species new to science.

## Materials and methods

In a total, five specimens (23 individuals) of the new species were collected from three adjacent localities. The illustrations of the species were made from dry materials by using Adobe Photoshop CS4. Herbarium studies were made in Akdeniz University Herbarium, which has the richest *Cephalaria* collections in Turkey, and ANK, GAZI and HUB.

## Taxonomic treatment

### Cephalaria anamurensis

Taxon classificationPlantaeDipsacalesCaprifoliaceae

Göktürk & Sümbül sp. nov.

urn:lsid:ipni.org:names:77155495-1

Figs 1 , 2

#### Diagnosis.

*Cephalaria anamurensis* is similar to *Cephalaria elmaliensis* Hub.-Mor. & V. A. Matthews and *Cephalaria speciosa* Boiss. & Kotschy. It can be distinguished from them by its rhizomatous growth, the lower stem leaves 35−55 × 3.5−4.5 cm, a globose capitula, involucral bracts that are ovate-orbicular to triangular-ovate, 3−7 × 2.5−6 mm, and completely blackish or blackish on the dorsal side and at the acute or subobtuse apex, receptacular bracts that are oblanceolate and blackish at an acuminate apex, and the involucel with long teeth 3−4 mm long and short teeth 1-1.5 mm long.

#### Type.

TURKEY, Mersin, Anamur, Anamur to Kazancı, Kırkkuyu, Bıçkıcı boğazı, 36°28'35"N; 032°44'11"E, 1784 m, steppe and stony places, 24 July 2015 *Göktürk 8018, Sümbül & Çıngay* (holotype: Akdeniz University Herbarium 3446!; isotypes: ANK!, GAZI!, HUB!, NGBB!).

#### Description.

Plant stout, erect, perennial, rhizomatous herbs, up to 1.5 m, simple, striate, covered with densely stellate hairy and retrorse hairy in lower part. Leaves coriaceous, densely stellate hairy; lower stem leaves simple, lanceolate, 35−55 × 3.5−4.5 cm, crenate-serrate, acuminate; cauline leaves simple or lyrate; simple leaves lanceolate or broadly lanceolate, 10−30 × 1.5−5.5 cm, entire, acuminate; lyrate leaves lanceolate or broadly lanceolate in outline, 10−25 × 2−5 cm, with 2−3 segments, lateral segments lanceolate, 0.3−1.5 × 0.1−0.4 cm, entire, acute, terminal segment larger than lateral ones, lanceolate or broadly lanceolate, 8−22 × 2−5 cm, margins entire or crenate-serrate, acute; upper stem leaves simple, sessile, linear or linear-lanceolate, 0.5−6 × 0.2−0.8 cm, entire, acuminate. Capitula globose, 25−50-flowered, 2−4 cm in diameter in flower, 2−3 cm in diameter in fruit. Involucral bracts ovate-orbicular to triangular-ovate, 3−7 × 2.5−6 mm, completely blackish or blackish in dorsal side and at apex, pubescent or adpressed pilose, margins ciliate, acute or subobtuse at apex; receptacular bracts oblanceolate, 8−13 × 2−4 mm, straw-coloured on dorsal side and base, blackish at apex, pubescent and adpressed pilose in dorsal side and apex, margins ciliate, acuminate at apex. Calyx cupuliform, 1−2 mm in diameter, with irregular teeth. Corolla cream or pale yellow, 8−15 mm long, densely adpressed hairy outside. Involucel 4-angled, 7−13 mm long in fruit, pilose, 4 long and 4 short teeth at apex; long teeth 3−4 mm long, short teeth 1−1.5 mm long. Flowering from July to August, fruiting from August to September.

#### Distribution and ecology.

**Figure 1.**
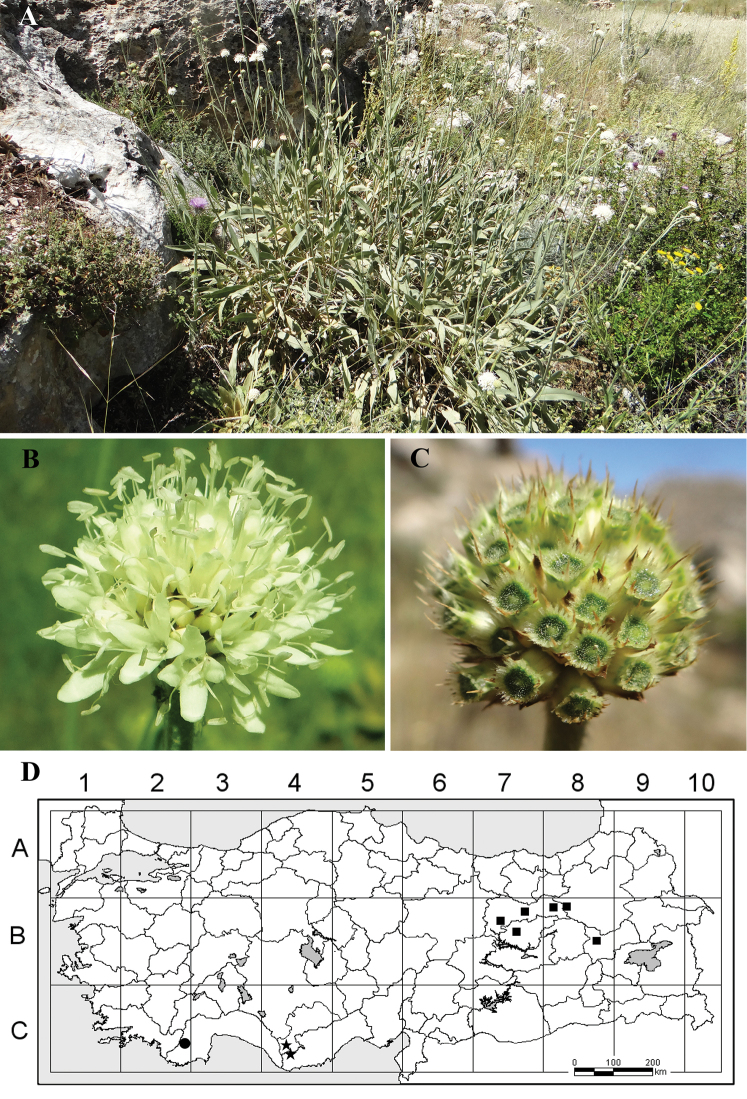
Photographs and distribution map of *Cephalaria anamurensis*. **A** habit and habitat of the type plant **B** Close-up of flowering capitula **C** Close-up of fruiting capitula **D** Distribution of *Cephalaria anamurensis* (★), *Cephalaria elmaliensis* (●) and *Cephalaria speciosa* (■) in Turkey. Photos: Ramazan Süleyman Göktürk.

This species is endemic to South Anatolia, Turkey, and east Mediterranean (mountain) element (Fig. 1D). According to EUNIS (2007), habitat type of this new species is E2.5 (Meadows of the steppe zone). *Cephalaria anamurensis* grows in steppe and stony places at an elevation of 1784−1800 m. It is associated with endemics such as Marrubium lutescens Boiss. & Heldr. subsp. micranthum (Boiss. & Heldr.) P. H. Davis, Nepeta nuda L. subsp. glandulifera Hub.-Mor. & P. H. Davis, Papaver pilosum Sibth. & Sm. subsp. pilosum, Sideritis libanotica Labill. subsp. violascens (P. H. Davis) P. H. Davis, *Verbascum cucullatibracteum* Hub.-Mor. and non-endemic plants such as Digitalis ferruginea L. subsp. ferruginea, *Euphorbia kotschyana* Fenzl, *Berberis crataegina* DC., *Genista albida* Willd., *Hordeum bulbosum* L., *Onopordum sibthorpianum* Boiss. & Heldr., *Phlomis armeniaca* Willd., Scutellaria orientalis L. subsp. pinnatifida J. R. Edm. and *Thymus sipyleus* Boiss.

**Figure 2.**
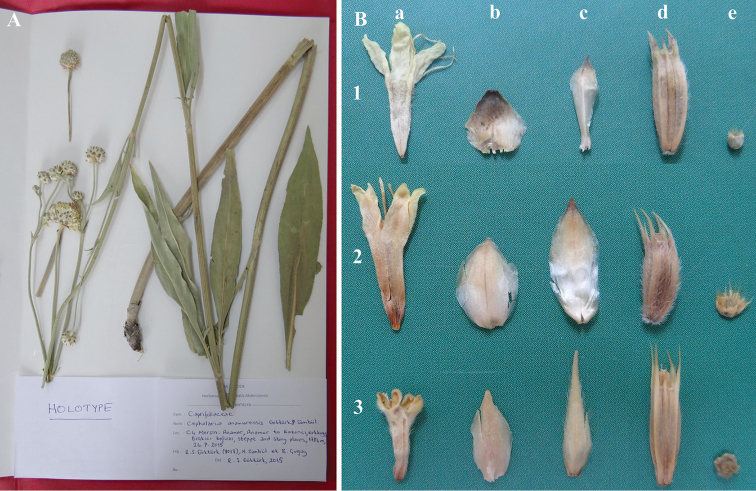
**A** Holotype specimen of *Cephalaria anamurensis* Göktürk & Sümbül **B** Reproductive parts of *Cephalaria anamurensis* (*Göktürk 8018*) (**1**), *Cephalaria elmaliensis* (*Göktürk 3532*) (**2**) and *Cephalaria speciosa* (*Göktürk 4727*) (**3**): **a** corolla **b** involucral bract **c** receptacular bract **d** involucel **e** calyx.

#### Conservation status.

This species is known only from three adjacent localities with small populations in Kırkkuyu collected by the authors. It is suggested that this new species should be placed under the IUCN threat category “Critically Endangered (CR)” (IUCN 2014) because the estimated area of occupancy is less than 10 km^2^, the population size of the new species is estimated to be less than 50 mature individuals, and the population size of the new species could be reduced in the near future based on heavily grazing pressure [CR B2; C2a(i)].

#### Etymology.

The specific epithet is derived from the name of Anamur district in Mersin province, where the holotype of *Cephalaria anamurensis* was collected.

## Results

The new species is included in a group of *Cephalaria* species that are covered with stellate hairs. There are nine species in this group in Turkey, and eight of them are endemic to the country. Only *Cephalaria stellipilis* Boiss. has a distribution extending out of Turkey to Lebanon. *Cephalaria anamurensis* is morphologically closest to *Cephalaria elmaliensis* and *Cephalaria speciosa*. *Cephalaria elmaliensis* is endemic to Çığlıkara nature protection area and grows on stony ground and in openings in cedar forests (Cedrus libani A. Rich. var. libani) in the Elmalı district/Antalya. *Cephalaria speciosa* is endemic to east Anatolia and grows in rocky places and roadsides (Fig. 1D). A comparison of *Cephalaria anamurensis*, *Cephalaria elmaliensis* and *Cephalaria speciosa* is given in Table 1.

**Table 1.** Morphological comparison of *Cephalaria anamurensis*, *Cephalaria elmaliensis* and *Cephalaria speciosa*.

Characters	*Cephalaria anamurensis*	*Cephalaria elmaliensis*	*Cephalaria speciosa*
Stem	rhizomatous, up to 1.5 m, retrorse hairy in lower part	not rhizomatous, up to 1 m, antrorse hairy in lower part	not rhizomatous, up to 1.5 m, retrorse hairy in lower part
Lower stem leaves	lanceolate, 35−55 × 3.5−4.5 cm, crenate-serrate, acuminate	lanceolate, 10−26 × 1.3−4 cm, entire or crenate-serrate, acute	oblong-lanceolate, 10−40 × 2.5−6.5 cm, entire or crenate-serrate, acute or acuminate
Lyrate cauline leaves	lanceolate or broadly lanceolate in outline, 10−25 × 2−5 cm, with 2−3 segments	narrowly ovate-lanceolate in outline, 6−15.5 × 0.8−1.6 cm, with 2−6 segments	lanceolate or oblong-lanceolate in outline, 8−20 × 3−6 cm, with 2−6 segments
Capitula	globose, 2−4 cm in diameter in flower	subglobose, 1−2 cm in diameter in flower	ovate to globose, 2.5−4.5 cm in diameter in flower
Involucral bracts	ovate-orbicular to triangular-ovate, 3−7 × 2.5−6 mm, completely blackish or blackish in dorsal side and at apex, acute or subobtuse	ovate, 4−6 × 2.5−3 mm, completely straw-coloured or only brown at apex, acute	ovate to triangular- lanceolate, 7−15 × 3−7 mm, completely straw-coloured, acuminate or subacuminate
Receptacular bracts	oblanceolate, 8−13 × 2−4 mm, blackish at apex, acuminate	ovate or lanceolate, 8−12 × 2−3 mm, completely straw-coloured, acute	triangular-lanceolate, 12−20 × 3.5−6 mm, brown at apex, pungent
Involucel	long teeth 3−4 mm long, short teeth 1-1.5 mm long	long teeth 3 mm long, short teeth 1 mm long	long teeth 4 mm long, short teeth 2 mm long

The genus *Cephalaria* was represented by 29 species in the *Flora of Turkey and the East Aegean Islands* (Matthews 1972). Since then, 11 new species, one new subspecies and one variety have been described from Turkey (Matthews 1988, Sümbül 1991, Göktürk and Sümbül 1997, Göktürk et al. 2003, Göktürk and Sümbül 2003, Kuş and Göktürk 2005, Aksoy et al. 2007, Parolly and Eren 2007, Göktürk et al. 2012). Göktürk and Sümbül (2014) placed *Cephalaria amana* Rech. f. as a synonym of *Cephalaria taurica* Szabó. According to Göktürk and Sümbül (2014), the total number of species of *Cephalaria* reported from Turkey is 39 and the total number of taxa of *Cephalaria* is 41 in Turkey. With the description here of *Cephalaria anamurensis* the number of species in Turkey is now 40 and the total number of taxa of *Cephalaria* is also 42 in Turkey.

A morphological key of *Cephalaria* species in Turkey with stellate hairs.

**Table:** 

1	Lower stem leaves lyrate	**2**
–	Lower stem leaves simple	**4**
2	Lower stem leaves only with sparsely stellate hairs, stem hollow	***Cephalaria demirizii***
–	Lower stem and cauline leaves with dense stellate hairs, stem not hollow	**3**
3	Bracts blackish at apex; involucral bracts acute or subacute; receptacular bracts ovate-oblong	***Cephalaria davisiana***
–	Bracts straw-coloured at apex; involucral bracts obtuse; receptacular bracts oblong or narrowly oblanceolate	***Cephalaria sumbuliana***
4	Petiole of lower stem leaves deflexed	***C. duzceënsis***
–	Petiole of lower stem leaves not deflexed	**5**
5	Capitula ovoid; involucel sericeous	***Cephalaria elazigensis***
–	Capitula not ovoid; involucel pilose	**6**
6	Plant stout, greater than 1 m high	**7**
–	Plant slender, up to 1 m high	**8**
7	Capitula ovate-globose; involucral bracts ovate to triangular-lanceolate	***Cephalaria speciosa***
–	Capitula globose; involucral bracts ovate-orbicular to triangular-ovate	***Cephalaria anamurensis***
8	Lower and cauline stem leaves lanceolate	***Cephalaria elmaliensis***
–	Lower and cauline stem leaves oblong-spathulate	***Cephalaria stellipilis***

## Specimens examined

***Cephalaria anamurensis*** (Paratypes), TURKEY- C4 Konya: Ermenek, Kazancı district plateau, Kırkkuyu, 1800 m, 19.07.1984, *Sümbül 3217* (HUB!, ANK!); C4 Mersin: Anamur, Anamur to Kazancı, Kırkkuyu, Bıçkıcı boğazı, 36°28’31"N; 032°44'53"E, 1800 m, steppe and stony places, 24.07.2015, *Göktürk 8020, Sümbül & Çıngay* (Akdeniz University Herbarium!); C4 Mersin: Anamur, Anamur to Kazancı, Kırkkuyu, Bıçkıcı boğazı, 36°28'20"N; 032°45'05"E, 1800 m, steppe and stony places, 24.07.2015, *Göktürk 8024, Sümbül & Çıngay* (Akdeniz University Herbarium!); C4 Mersin: Anamur, Anamur to Kazancı, Kırkkuyu, Bıçkıcı boğazı, 36°28'20"N; 032°45'05"E, 1800 m, steppe and stony places, 29.09.2015, *Göktürk 8066 & Sümbül* (fruiting) (Akdeniz University Herbarium!).

***Cephalaria elmaliensis*** TURKEY- C2 Antalya: Elmalı, Çığlıkara, near security building, in openings in Cedrus libani var. libani, 1700−1900 m, 25.08.1993, *H. Duman et al. 5345* (GAZI!, Akdeniz University Herbarium!); C2 Antalya: Elmalı, Çığlıkara, near security building, in openings in Cedrus libani var. libani, 1700−1900 m, 12.08.1995, *Göktürk 3532* (Akdeniz University Herbarium!); Elmalı, Çığlıkara, between Ayıngediği-Kaş gediği, in openings in Cedrus libani var. libani, stony ground, 1750 m, 17.08.2007, *Göktürk 6111* (Akdeniz University Herbarium!).

***Cephalaria speciosa*** TURKEY- B7 Erzincan: Keşiş mountain, Cimin, rocky slopes, c. 2300 m, 28.08.1957, *P. H. Davis 31828* (ANK!); Kemah, above Kömürköy, steppe, 1850 m, 31.07.1996, *Dönmez 53*67 (HUB!); Tunceli: Ovacık, Munzur Mountain, Aksu stream, c. 1700 m, 21.07.1957, *P. H. Davis 31462 & I. C. Hedge* (ANK!); B8 Erzincan: Aşkale to Tercan, dry rocky places, c. 1700 m, 25.08.1957, *P. H. Davis 32657 & I. C. Hedge* (ANK!); between Tercan-Aşkale, slopes, 1765 m, 17.08.2000, *Göktürk 4528 & F. Göktürk* (Akdeniz University Herbarium!); B8 Muş: Varto, Muş to Varto, rocky slopes, 1350 m, 02.08.2001, *Göktürk 4727 & M. Göktürk* (Akdeniz University Herbarium!).

## Supplementary Material

XML Treatment for Cephalaria anamurensis
